# Healthcare Worker Perceived Barriers to Early Initiation of Antiretroviral and Tuberculosis Therapy among Tanzanian Inpatients

**DOI:** 10.1371/journal.pone.0087584

**Published:** 2014-02-14

**Authors:** Bahati M. K. Wajanga, Robert N. Peck, Samuel Kalluvya, Daniel W. Fitzgerald, Luke R. Smart, Jennifer A. Downs

**Affiliations:** 1 Department of Internal Medicine, Bugando Medical Centre, Mwanza, Tanzania; 2 Department of Internal Medicine, Catholic University of Health and Allied Sciences - Bugando, Mwanza, Tanzania; 3 Center for Global Health, Department of Medicine, Weill Cornell Medical College, New York, New York, United States of America; Institute of Infectious Diseases and Molecular Medicine, South Africa

## Abstract

**Setting:**

Clinical trials have shown that early initiation of antiretroviral therapy in HIV-infected patients with tuberculosis saves lives, but models for implementation of this new strategy have been under-studied in real-world settings.

**Objective:**

To identify the barriers and possible solutions for implementing concurrent early treatment with antiretroviral and anti-tuberculosis therapy in a large East African referral hospital where the prevalence of both infections is high.

**Design:**

In-depth interviews among hospital administrators, laboratory technicians, nurses, pharmacists, and physicians.

**Results:**

Twenty-six hospital staff identified six key barriers and corresponding solutions to promote rapid initiation of antiretroviral therapy in HIV-infected inpatients with tuberculosis. These include revising systems of medication delivery, integrating care between inpatient and outpatient systems, training hospital nurses to counsel and initiate medications in inpatients, and cultivating a team approach to consistent guideline implementation.

**Conclusion:**

Most barriers identified by hospital staff were easily surmountable with reorganization, training, and policy changes at minimal cost. Efforts to reduce mortality for HIV and tuberculosis co-infected patients in accordance with new World Health Organization guidelines are currently hampered by implementation barriers in real-world settings. Our findings suggest that these can be overcome with strategic enactment of simple, realistic interventions to promote early dual treatment for HIV/tuberculosis co-infected patients.

## Introduction

Recent studies have documented that HIV-positive patients with tuberculosis (TB) who receive antiretroviral therapy (ART) within two weeks of beginning anti-tuberculosis treatment have lower mortality, particularly at low CD4 counts [1]–[4]. These findings prompted the World Health Organization (WHO) to revise its guidelines in 2012, urging initiation of ART “as a matter of emergency (within two weeks of starting anti tuberculosis treatment)” in patients with CD4 counts <50 cells/µl and “as early as possible” in others.

Though the guidelines have now been in place for a year, numerous barriers have complicated their implementation, and many HIV/TB co-infected patients continue to die due to delayed initiation of concurrent treatment. For example, although the Tanzanian Ministry of Health has adopted these WHO guidelines for co-infected patients [5], our experience at a large Tanzanian referral hospital suggests that, in much of Tanzania, it is still common practice to complete TB treatment before initiating ART. Data from African and other resource-poor countries also suggests that 20–40% of patients who are not receiving ART at the time of TB diagnosis may remain without ART for at least the first few months of their six-month anti-TB treatment course [6]–[8].

We hypothesized that current hospital policies and practices pose challenges for early initiation of both therapies, and that these might be easily modifiable. We therefore sought to determine barriers to early initiation of ART and anti-tuberculosis therapy at Bugando Medical Centre, and to gain health workers’ insights for overcoming these barriers.

## Methods

### Study Site

This study was conducted at Bugando Medical Centre (BMC), the regional referral hospital for northwestern Tanzania. Located in the city of Mwanza, BMC’s adult medical wards admit ∼350 patients/month. Approximately 20% of adult medical admissions are HIV-positive, and ∼25% of HIV-positive inpatients have tuberculosis co-infection [9], [10]. BMC’s outpatient HIV clinic is the regional ART training center, providing care for 12,000 outpatients, of which 3,500 are receiving ART.

### Participants

BMC hospital staff were invited to participate, by convenience sampling, from a variety of inpatient and outpatient settings. Participants provided written informed consent.

### Interviewer

The principal investigator (B.W.) conducted all interviews. He is a medical doctor specialized in internal medicine. He has worked in the department of internal medicine at BMC for the past seven years, and he personally knows the majority of the study participants. He regularly attends medical patients on the medical wards and in the medical outpatient clinic. He would like to improve the care of TB and HIV co-infected patients and chose to complete a qualitative study to explore barriers to their care.

### Data Collection and Analysis

In-depth, one-on-one interviews lasting ∼20 minutes were conducted in Kiswahili or English depending on the participant’s preference. The principal investigator (B.W.) used a structured questionnaire. Interviews were recorded, transcribed into Kiswahili, and translated into English by a professional service in Mwanza.

The goal was to explore themes [11] surrounding barriers to prompt, early initiation of both ART and anti-TB for inpatients. In particular, efforts were made to explore participants’ opinions on issues rather than to explain issues objectively [12]. First, the study team discussed transcripts to determine broad themes. Two investigators then coded data independently using NVivo software (Version 10, QSR International, Doncaster, Australia). Themes identified by both researchers were organized and supporting quotations were selected. Themes not identified by both were presented to other team members for a final decision about inclusion.

This study was approved by the BMC ethics committee and Weill Cornell Medical College’s Institutional Review Board.

## Results

From March-June 2012, we interviewed 26 hospital workers: ten inpatient physicians, two HIV outpatient physicians, three inpatient nurses, three laboratory technologists, two administrators, two HIV outpatient nurses, one inpatient pharmacist, and one HIV clinic pharmacist. Staff identified six key barriers and recommended potential solutions. These are summarized in **Table 1** and discussed below.

**Table 1:** Key Barriers and Solutions to Implementing Prompt and Concurrent HAART and Anti- Tuberculosis Therapy Identified by Bugando Medical Centre Staff Members in Mwanza, Tanzania.

Barriers	Solutions
Physical unavailability of medications for inpatients – ART and anti-TB medications are considered outpatient drugs and are kept in separate outpatient pharmacy	•Make ART and anti-TB medications available in inpatient pharmacy
	•Place emergency supply of medications “like PEP” (post-exposure prophylaxis) in wards
Poor integration between inpatient and outpatient HIV and TB careleading to unavailability of some essential services for inpatients	•Train inpatient nurses to perform traditionally outpatient jobs (medication counseling, ART training sessions, medication distribution)
Requirement for three counseling sessions to be completedprior to initiation of ART	•Train inpatient nurses to perform counseling
	•Ease policy to allow concurrent counseling with medication administration
Shortage of staff during weekends and holidays	•Create responsible on-call team (“like PEP”) for TB and ART medication administration
	•Train inpatient nurses
Lack of awareness and/or acceptance of new guidelines forearly ART initiation	•Continuing education for inpatient nurses, pharmacists, physicians, and other healthcare providers
Patients’ fear of stigma may lead those with TB not todisclose their HIV status	•Cultivation of positive multidisciplinary team approach to patient care
	•Partnership with treatment partners and peer counselors

Key: ART: antiretroviral therapy. HAART: highly active anti-retroviral therapy. PEP: post-exposure prophylaxis. TB: tuberculosis.

### Physical Unavailability of Medications for Inpatients

A barrier frequently reported by both pharmacists and physicians is the physical unavailability of anti-tuberculosis medications and ART for inpatients. Unlike other medications, anti-tuberculosis medications and ART are dispensed from their segregated outpatient pharmacies and not from the inpatient pharmacy:

*Currently Anti-TBs are centralized–they have their own system of issuing to patients. They are not issued as other drugs that we issue from the pharmacy department. The same applies to antiretroviral drugs– they have got their own clinic.* (Inpatient pharmacist).

*We don’t come in contact with patients who are admitted.* (Outpatient pharmacist).

#### Solution

Physicians, pharmacists, and nurses urged making all medications available in the wards:

*I would advise [that]… the supply of Anti-TB drugs should follow the same route as the common drugs so that…patients who are admitted should receive drugs straight from the pharmacy and from the wards.* (Inpatient pharmacist)

*Like what we are doing with PEP [Post-Exposure Prophylaxis], those medications are stored in the ward, in case of emergency are given to people who are eligible, so we should do for ART as well.* (Nurse)

### Poor Integration Between Inpatient and Outpatient HIV and TB Care

Nearly all respondents explained how un-integrated HIV and TB services delay and lessen the quality of care for co-infected patients. TB-related care for all TB patients, whether inpatients or outpatients, is coordinated by two outpatient-focused “TB nurses.”

*Only two nurses…serve the whole hospital including the [HIV] clinic. They are responsible in storage, administering and dispensing of the drugs.* (Physician)

*TB services are handled by somebody who works as an outpatient care provider.* (Physician)

Similarly, ART initiation is seen as an outpatient activity, and educational counseling that is required prior to the initiation of ART is performed by outpatient HIV nurses. Inpatient initiation of ART requires physicians to *“consult the [outpatient HIV clinic] team to come and do post-test counseling, do the register then and probably start them on ART”* but is hindered because *“sometimes this person…has some delay”* (Physician). Therefore, initiation of ART in inpatients is unusual:

*Most of the patients we discharge them through [the HIV clinic]. They are not started immediately in the ward.* (Physician)

*It is not an easy task to initiate ART in the wards.* (Physician)

#### Solution

Staff uniformly suggested integrating inpatient and outpatient HIV/TB-related services rather than treating the two infections separately:

*I would advise if possible that we integrate the inpatient department and the tuberculosis services to be in one department so is the TB and HIV clinics integrated.* (Physician)

*We have separate entities…of management–TB being treated separately and HIV being also treated separately–but now we are seeing this problem as becoming more together than living separately so we need to treat them together.* (Administrator)

Another specific suggestion was to train inpatient nurses as ART/anti-TB medication counselors so that inpatients do not have to wait for outpatient staff to come to their bedside or travel from their hospital bed to the outpatient clinic:

*If you want to start this protocol…you have to involve all nurses not just those who are at TB clinic…I doubt if all the nurses apart from these two have the required knowledge like…which medication they…are supposed to use, and how they will start these medications.* (Physician)

*[We should] have a counselor at least in each ward doing the adherence counseling. For the admitted patient to go to clinic it may take time, more than a week.* (Physician)

### Requirement for Multiple Pre-ART Counseling Sessions

Adherence counseling among HIV clinic outpatients consists of week-long classes, beginning on Mondays. Outpatients are given ART on Fridays after completing these sessions and successfully passing an examination. Healthcare workers described the challenges and delays encountered when trying to perform this adherence counseling for inpatients:

*It depends if the counselor will be available for example soon after the diagnosis. It is not taking long but the problem is if the counselor will not be available that time then patients may stay in the ward for more than 2–3 weeks waiting for the adherence counseling.* (Physician)

#### Solution

Respondents again suggested training inpatient nurses to perform adherence counseling to expedite ART initiation:

*I would [suggest] training the nurses in the wards to give adherence sessions especially in the initial part of the adherence session so the ball starts rolling as soon as the patient is admitted.* (Physician)

*Counseling may not only be done during the weekdays but also…during the weekends and maybe…not only three days in a week. Maybe if they can do…for those who came on Wednesday for example they can start even on Thursday.* (Physician)

### Staff Shortages During Weekends/Holidays

A shortage of staff able to initiate ART and anti-TB medications was seen as particularly problematic during weekends and holidays. Hospital staff accepted as a given that patients admitted during these time periods could not begin ART or anti-TB medications until weekdays:

*If a patient is admitted in the ward… during weekends and public holidays those who are responsible for issuing…Anti-TBs are not available within the hospital…the current system denies patients access to these drugs during weekends.* (Pharmacist)

*[The outpatient HIV clinic] providing drugs is not working on weekends.* (Physician)

#### Solution

Physicians, nurses, and administrators agreed on the necessity of additional weekend staff and suggested assigning responsibility so that WHO guidelines could be implemented at all times:

*[We] need to address the hospital management so that they can revise the system of allocating…who is the responsible team even in the weekend.* (Physician)

*I think just like what we do with PEP that we should have people responsible during weekends and holidays for initiation like every time all the time.* (Nurse)

### Lack of Awareness/Acceptance of Guidelines

We found that many pharmacists, nurses, and physicians were not aware of the new WHO guidelines, although they have been adopted by Tanzania. Others admitted knowing the new guidelines but being reluctant to implement them out of fear of complications. Commonly-mentioned complications were immune reconstitution inflammatory syndrome (IRIS), drug interactions, and heavy pill burdens:

*These patients are seriously ill and taking both regimes at the same time may bring unnecessary burden to them…Another thing is the probability of having drug interactions between anti-tuberculosis and ART.* (Pharmacist)

#### Solution

Participants stated that providing staff with continuing education and training about the new guidelines and supporting data would facilitate their acceptance and implementation.

*The first challenge is awareness of the advantages of combining both therapies for such patients, I think, so the more aware we people are the better it will be to implement this protocol.* (Physician)

*We are getting new doctors especially interns every time. Updating when new information arises or when changing protocols happens is very important for proper care of patients.* (Nurse)

### Delays Due to Patients Concealing their HIV Statuses

Even with the implementation of provider-initiated HIV testing and counseling as hospital policy in Tanzania, testing is still not routine for all inpatients and many avoid being tested. A nurse explained that even patients who do know their HIV status may not be forthcoming:

*Those patients who are admitted to hospital different [from where] they were getting HIV care usually delay on revealing that they are HIV-positive…and may tell only one person and insist that it should be a secret.*

#### Solution

Diagnostic and treatment delays due to patients’ unknown HIV statuses were regarded by hospital staff as a problem that could be overcome by cultivating a positive inpatient environment. Participants stated that cohesiveness and non-judgmentalism among hospital staff would engender patients’ trust and ease their fears of being stigmatized:

*Having team of nurses and doctors giving all service to the patients who are admitted will win confidence of these patients and therefore [they] will be open.* (Nurse)

More generally, creating a team environment among hospital workers was perceived as essential to successful implementation of the new guidelines. Healthcare workers were enthusiastic and hopeful about their potential to achieve new and better standards of patient care through education and teamwork:

*I believe that it’s all about information like if everyone…all the nurses all the doctors all the pharmacists will be involved in this and informed that we are going to start this and everyone will be in the same page I think it can be done and it’s possible.* (Physician)

## Discussion

Qualitative interviews with a variety of hospital staff in Tanzania revealed six largely-surmountable barriers to early implementation of both antiretroviral and anti-tuberculosis therapy in inpatients. Three barriers–poor inpatient/outpatient and TB/HIV integration, lack of trained staff during weekends, and lack of awareness and/or acceptance of new WHO guidelines–can be addressed through continuing education for healthcare workers. Two additional barriers–unavailability of anti-tuberculosis and antiretroviral medications in the inpatient pharmacy, and an inflexible system regarding ART counseling sessions–can be resolved through revisions to hospital policy. The sixth barrier, patients’ reluctance to disclose their HIV status, can be improved with a positive team-oriented approach to patient care that fosters patients’ trust. Our data suggests that simple, practical strategies to educate healthcare workers and modify institutional policies could substantially increase implementation of the WHO guidelines, which have a proven mortality benefit for HIV/TB co-infected patients.

Despite rapid advances in diagnostic and therapeutic management of HIV in sub-Saharan Africa over the past ten years, our findings highlight that the framework for HIV care provision that was laid down early in the epidemic remains unchanged in many healthcare settings. Multiple early ART treatment programs, created rapidly in response to an emergency, were modeled after existing vertical TB treatment programs. Analogous to TB control efforts, “vertical stewardship and governance” of HIV care was believed to facilitate HIV surveillance, quality assurance, and coordination at a national level [13]. Such vertical constructs of HIV care provide tighter control but preclude collaborative management of patients who, in addition to HIV, suffer from other conditions. More streamlined, integrated HIV care that permits management of multiple health issues by one team is essential to optimizing care of HIV-infected patients and to operationalizing the 2012 WHO guidelines for cooperative HIV/TB activities [14], [15].

Shifting towards integrated programs has been the focus of numerous efforts in sub-Saharan Africa and other regions with heavy burdens of both HIV and TB [13], [16], [17]. Our work reveals that many barriers hinder integration of HIV/TB care in the inpatient setting. For example, as noted by many hospital staff, pre-ART counseling has typically been performed by outpatient nurses, and only by specially-trained nurses. Similarly, our study’s finding that the standard medications for treatment of classically “outpatient” infections–HIV and TB–are not available on inpatient wards reveals further need for operational improvements that will increase the accessibility of medications for inpatients who urgently need them.

Our work additionally reflects the need for a multidisciplinary approach to HIV care now that wide spread ART has transformed HIV infection into a chronic condition. Whereas previously some healthcare providers specialized in HIV alone, HIV-infected patients now present with diverse co-morbidities requiring complex management. Therefore, the model of providing HIV care through an independent program that is segregated from standard primary care, as done in many sub-Saharan countries including Tanzania [5], is obsolete. Lack of integration also limits familiarity with HIV management to the few healthcare providers who see these patients in outpatient clinics.

Another traditional practice that many hospital staff viewed as outdated is requiring completion of multiple counseling sessions before patients are permitted to initiate ART. This requirement may be a vestige of decades-old fears of donor nations that poor people “without watches” could not adhere to ART, which led to mandated intensive pre-treatment counseling. While patient non-adherence is still associated with higher risks of mortality [18], more recent research suggests that patients should be initiated on ART rapidly and undergo supportive counseling as they begin ART rather than waiting to complete training [19]. We suggest that, particularly for inpatients whose mortality is high due to concurrent disease, initiation of potentially life-saving ART should not be delayed by multiple training courses. Additional healthcare workers should be taught to perform, or at least begin, training sessions so that critical patients’ ART is not delayed.

Our finding that many hospital staff were reticent to implement the new WHO guidelines for HIV/TB co-infected patients is concerning. Lack of knowledge has similarly been identified as a barrier to implementation of optimal TB/HIV care in other studies in Tanzania and Uganda [19], [20]. Importantly, staff members in our study admitted believing that risks of IRIS, drug interactions, or pill burdens could outweigh the benefits of early ART in some patients. This highlights the need for targeted education that discusses proven mortality benefits and encourages staff to put nationally- and internationally-accepted evidence ahead of personal beliefs.

Our work is a single-center study that highlights the need for additional research in this area, both in our setting and in other resource-poor regions. First, it will be crucial to confirm whether the barriers perceived by healthcare workers are actual, objective barriers to implementation of WHO guidelines. It will also be important to assess perceptions of barriers from patients’ perspectives. Future studies will subsequently need to determine whether these recommended solutions, once implemented, effectively lessen both perceived and actual barriers. Such ongoing research is imperative for successful operationalization of the WHO guidelines.

## Conclusions

Integration of HIV and TB care is vital for optimal enactment of WHO guidelines. Our work highlights simple, practical ways in which care for HIV/TB co-infected inpatients can be streamlined. These strategies can counteract barriers to implementation among inpatients, leading to earlier treatment and lives saved.
